# Retrospective single-center historical comparative study between proGAV and proGAV2.0 for surgical revision and implant duration

**DOI:** 10.1007/s00381-022-05490-y

**Published:** 2022-03-30

**Authors:** E Brunner, A Schaumann, V Pennacchietti, M Schulz, UW Thomale

**Affiliations:** Present Address: Pediatric Neurosurgery, Charité Universitaetsmedizin Berlin, Campus Virchow Klinikum, Augustenburger Platz 1, 13353 Berlin, Germany

**Keywords:** Hydrocephalus, Cerebrospinal fluid, Shunt dysfunction, Adjustable valve, Overdrainage

## Abstract

**Objective:**

Cerebrospinal fluid (CSF) diversion shunt systems remain to be the most common treatment for pediatric hydrocephalus. Different valve systems are used to regulate CSF diversion. Preventing complications such as occlusions, ruptures, malpositioning, and over- or underdrainage are the focus for further developments. The proGAV and proGAV2.0 valve system are compared in this retrospective study for revision-free survival and isolated valve revision paradigms.

**Methods:**

In the first part of the study, the shunt and valve revision-free survival rates were investigated in a retrospective historical comparison design for a period of 2 years in which each valve was used as standard valve (proGAV: July 2012–June 2014; proGAV2.0: January 2015–December 2016) with subsequent 30-month follow-up period, respectively. In the second part of the study, the implant duration was calculated by detecting isolated valve (valve-only) revisions together with another valve explantation during the entire period of the first study and its follow-up period.

**Results:**

Two hundred sixty-two patients (145 male and 117 female, mean age 6.2 ± 6.1 years) were included in the cohort of revision-free survival. During the 30-month follow-up period, 41 shunt revisions, including 27 valve revisions (shunt survival rate: 72.1%, valve survival rate: 81.6%) were performed in the proGAV cohort and 37 shunt revisions, including 21 valve revisions (shunt survival rate: 74.8% and valve survival rate: 85.0%) were performed in the proGAV2.0 cohort without showing statistically significant differences. In the second part of the study, 38 cases (mean age 4.0 ± 3.9 years) met the inclusion criteria of receiving a valve-only-revision. In those patients, a total of 44 proGAV and 42 proGAV2.0 were implanted and explanted during the entire study time. In those, a significantly longer implant duration was observed for proGAV (mean valve duration 961.9 ± 650.8 days) compared to proGAV2.0 (mean length of implantation period 601.4 ± 487.8 days; *p* = 0.004).

**Conclusion:**

The shunt and valve revision-free survival rates were found to be similar among the groups during 30 month follow-up. In patients who received “valve only” revisions and a subsequent explanation, the implant duration was significantly longer in the proGAV. Although the amount of patients with valve-only-revisions are small compared to the entire cohort certain patients seem to be at higher risk for repeated valve revisions.

## Introduction

Pediatric hydrocephalus is one of the most common surgically treatable neurological conditions in children [1]. A cerebrospinal fluid (CSF) diversion shunt system is most often used for treatment [2, 3], which showed limited improvements over the past decades. Nevertheless, valve designs have become more sophisticated to address individual treatment possibilities by adjustability in order to avoid long-term over-drainage complications by implementing anti-hydrostatic components [4, 5]. This principle goal is to create a physiological-like level of CSF drainage, adjustable for the patients’ age and physical condition. However, further improvements will still be necessary in this regard.

Possible complications of CSF shunt treatment include infection, malpositioning, rupture, under-, and overdrainage. While the first four factors are linked to either surgically associated complications or shunt malfunction, overdrainage is a chronic issue linked to long-term inadequate CSF diversion. Constant overdrainage is associated with too low or even negative intracranial pressure leading to long-term consequences such as collapsed ventricles, risk of ventricular catheter obstruction, slit ventricle syndrome, subdural hematomas, bone hyperplasia, microcephaly, and shunt-induced Chiari malformation [6–14]. Possible reasons for overdrainage are the hydrostatic effect in the upright position and increasing activity of the patients associated with relevant differential pressure changes between the peritoneal and intracranial cavity.

Underdrainage is often related to any obstruction of the shunt system, which might be occurring in the ventricular catheter, the valve or the peritoneal catheter as common reasons to undergo shunt revision procedures. Specifically, valve occlusions are associated with accumulation of proteins in the valve’s lumen over time impairing the functional unit of adjustment or anti-hydrostatic mechanism or finally leading to blockage of CSF flow [5, 14, 15].

**Fig. 1:**
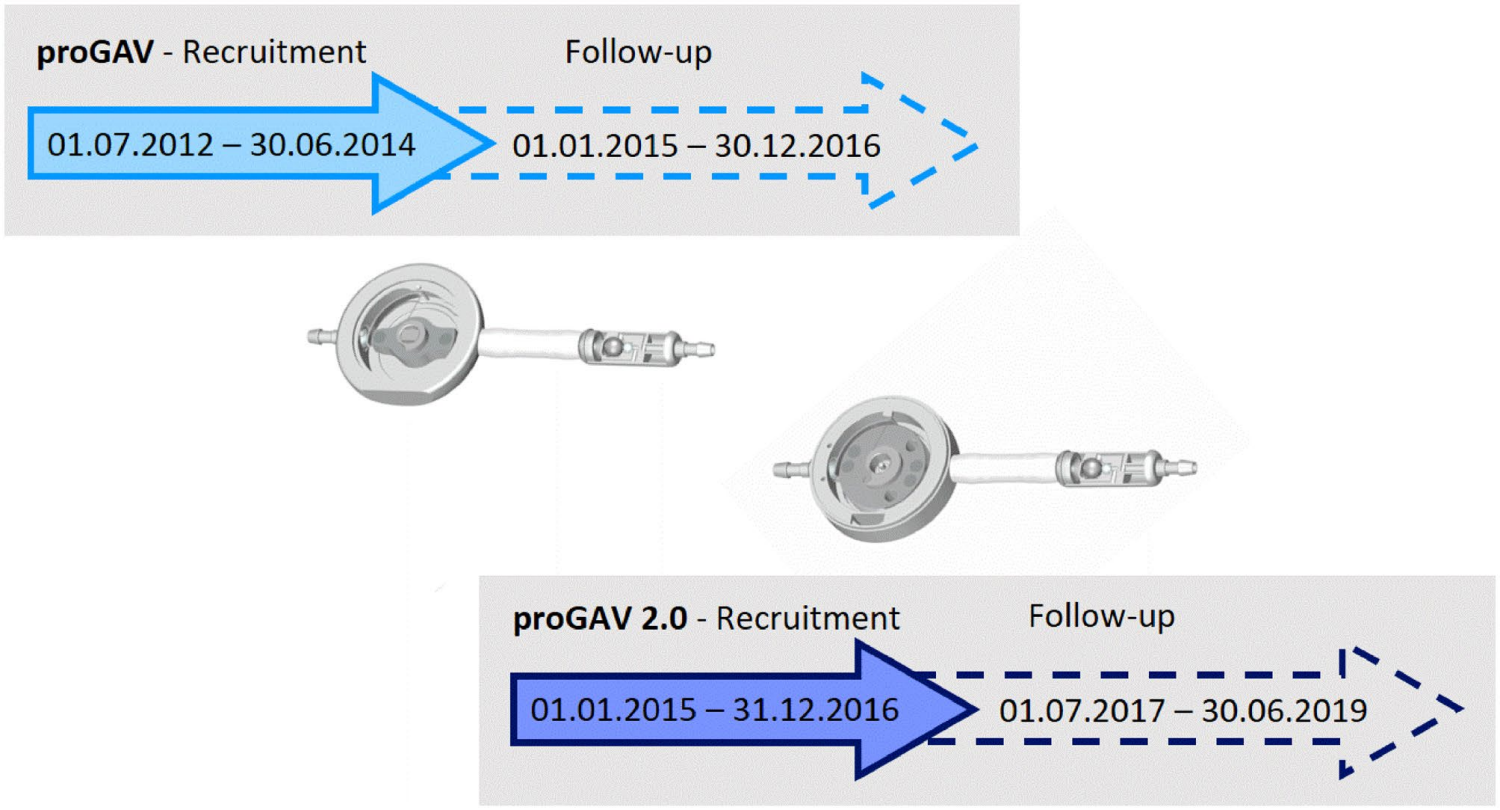
First study part: Inclusion criteria were valve implantation took place during a 2-year period in which either proGAV (7/2012–6/2014) or proGAV2.0 (1/2015–12/2016) was used as a standard valve, respectively. Surgical shunt revision was detected in this cohort during an individual 30-months follow-up period

Among various models of valves available on the market, in our center, a strict regimen was implemented since 2007 to use adjustable differential pressure valves with an anti-hydrostatic functionality in all pediatric hydrocephalus cases with shunt dependency. With this preconditions mainly proGAV or proGAV 2.0 (Miethke, Aesculap, Potsdam, Tuttlingen, Germany) have been used, which consists of a programmable differential pressure valve (0–20 cmH_2_O) and a gravity-assisted unit adding further resistance in vertical body position (usually 20 or 25 cmH_2_O; Fig. 1) [16–18]. The proGAV 2.0 valve represents a successor model to the proGAV valve in order to enable easier adjustability function. The reduced lumen inside the proGAV2.0 valve was designed to increase intra-valve CSF flow velocities and reducing the so called dead space of low- or no-flow regions with the aim to inhibit accumulation of protein deposits [4, 6, 19]. The proGAV 2.0 was started to be used since 2014 in our institution. Until today, only limited data was published to report the experience with the new valve design. Thus, we performed a single-center retrospective study aiming to investigate the experience with both valve models, the proGAV and proGAV2.0 as a historical comparison study to evaluate revision-free survival and specifically look for valve malfunction characteristics.

## Methods

In the single center, retrospective historical comparison study the shunt and valve revision-free survival rates on the one hand, and mean length of valve implant duration on the other hand, were investigated in two study parts.

In the *first study part*, the shunt and valve survival rates for proGAV and proGAV2.0 shunts were investigated within a comparable defined time period. Therefore, the respective time-periods of 2 years were selected in which either the proGAV or the proGAV 2.0 valve system was used as a primary standard valve. Thus, July 2012 to June 2014 was selected for the proGAV and January 2015 to December 2016 for the proGAV 2.0. These time periods were added by an individual follow-up period of 30 months, respectively (Fig. 1). Only cases were included who received a shunt surgery in our department with implantation of the above mentioned valve systems and who covered the entire follow up time, accordingly. Revision-free shunt survival was defined as an uneventful follow-up period without revision surgery for any part of the system, while revision-free valve survival was defined as an uneventful follow-up period without valve exchange. The Kaplan–Meier survival analysis as well as the total number of revision surgeries per case were evaluated and compared between the two valve groups. In addition, subgroup analyses were performed regarding age (infants versus non-infants) and diagnosis (post-hemorrhagic hydrocephalus (PHHC) versus other diagnosis).

In the *second study part*, the individual implantation time (implant duration) of the two valve models was analyzed. For this purpose, the entire study period between July 2012 and June 2019 was evaluated. The primary inclusion criteria were cases who underwent an isolated valve replacement (valve-only-revision) without any further changes in the shunt system at the time of surgery in combination with at least another valve exchange during follow up (1st valve revision). Thereby, the valve implant duration could be determined.

**Fig. 2:**
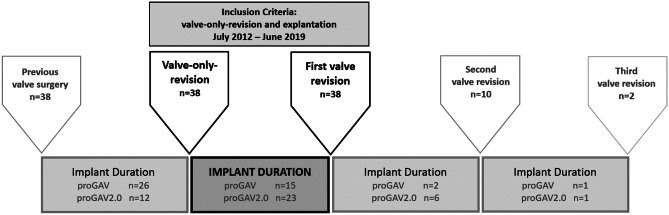
Second study part: inclusion criteria were a valve-only-revision detected between July 2012 and June 2019 together with an additional valve revision in order to calculate the implant duration. In this cohort of patients further previous and possible later valve revisions of the valve were investigated to further enhance the number of implant duration for better comparison

In order to calculate additional implant durations in this cohort of patients the previous as well as possible subsequent valve exchange surgeries were identified during the defined study-period. As shown in Fig. 2, the implant duration between two surgeries were recorded individually. Implant durations were evaluated for the proGAV and proGAV2.0 for comparison, accordingly.

### Statistical analysis

Excel (Microsoft Office, Alberquerque, NY, USA) was used to collect and organize the data. The graphical illustrations and Kaplan–Meier curves were created with SPSS 27 (IBM, Armonk, NY, USA). Other graphs were designed by Prism (GraphPad, San Diego, CA, USA). The Mantel-Cox test (log-rank test) was used to analyze the Kaplan-Meyer curves statistically and the chi-square test was used to examine the distribution of diagnosis. The two-tailed Mann Whitney test for unpaired results was used for the valve implantation time. A *p* value less than 0.05 was considered statistically significant. All values are given as mean ± standard deviation.

## Results

### Patient characteristics

#### First study part

The inclusion criteria met in 262 cases (145 male and 117 female; Table 1). As individual patients were operated more than once during the follow-up, a total number of 294 surgeries were detected. The mean age at the time of the valve implantation surgery was 6.2 ± 6.1 years (range: 0 to 28.8 years). Since the revision-free survival was calculated for the implants, respectively, 22 patients were represented in both the proGAV and the proGAV 2.0 subgroups but did not intervene among the groups. The primary etiology of hydrocephalus was post-hemorrhagic (*n* = 96; 37%), myelomeningocele (*n* = 65; 25%), tumor (*n* = 15; 6%), aqueductal stenosis (*n* = 14; 5%), post-infectious (*n* = 11; 4%), and other (*n* = 47; 18%). In 14 (5%) cases, it was not possible to retrospectively verify the original cause of hydrocephalus.

**Table 1:** Patient characteristics for the first group of patients

	**proGAV**		**proGAV 2.0**	
**Time period**	July 2012–June 2014		January 2015–December 2016	
**Number of patients** **(*n* = 262)**	142		142	
**Age** **6.2 ± **6.1	5.7 years ± 6.1 years (min: 0.0 years, max.: 28.5 years)		6.8 years ± 6.2 years (min: 0.0 years, max.: 28.8 years)	
Sex **m = 145 w = 117**	f: 72 m: 70		f: 58 m: 84	
**Number of operations** **(*n* = 294)**	147		147	
	Primary implants.: 49	Revision procedures: 98	Primary implants: 46	Revision procedures: 101
**Shuntsurvival rate** **at 30 months**	106 (72.1%)		110 (74.8%)	
**Valve survival rate** **at 30 months**	120 (81.6%)		125 (85.0%)	
**Revision surgeries**	41		37	
- **Ventricular catheter**	12		13	
- **Ventricular catheter and valve**	3		4	
- **Valve**	11		10	
- **Valve and distal catheter**	5		0	
- **Distal catheter**	2		3	
- **Complete shunt**	4		3	
- **Explantation**	4		4	

#### Second study part

**Fig. 3:**
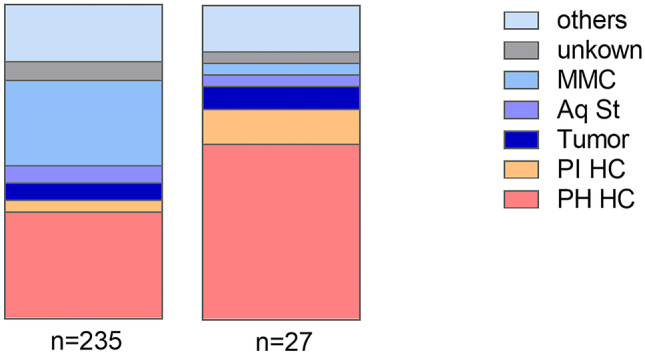
Comparison of the initial diagnosis leading to hydrocephalus of both study cohorts. The right column represents the 2nd study group (*n* = 27) while in the left column the remaining patients from the 1st study cohort are represented (*n* = 235). PHHC is the most common diagnosis in both groups, however less represented in the first group (PHHC: 34%) compared to the second study cohort (PHHC: 55%; *p* = 0.03). Abbreviations: Aq St = aqueductal stenosis, MMC = myelomeningocele, PHHC = post-hemorrhagic hydrocephalus, PIHC = post-infectious hydrocephalus

To calculate the implant duration, the inclusion criteria were valve-only-revision plus another valve exchange. These did meet in 27 (17 male and 10 female) cases from study 1. In addition, in 11 patients the surgery took place in the follow-up period of the first study period (Fig. 2). The mean age at the valve-only-revision was 4.0 ± 3.9 years (range: 0.3 to 17.2 years), and at the first, follow-up surgery 6.5 ± 4.5 years (range: 1.1 to 20.6 years). Twenty-eight patients (73.7%) had no further valve revision after the first follow-up operation, 8 patients (21.1%) had a total of two follow-up operations, and 2 patients (5.3%) had three follow-up operations. Similarly to the first part of the study, post-hemorrhagic hydrocephalus was the most common diagnosis (55.6%) but significantly more often represented (*p* = 0.03; Fig. 3). Other etiologies were post-infectious (11%), aqueductal stenosis (3.7%), tumor (7.4%), myelomeningocele (3.7%), and other (14.8%). In one case, the original cause of hydrocephalus remained unknown.

### Shunt and valve survival rates

**Fig. 4:**
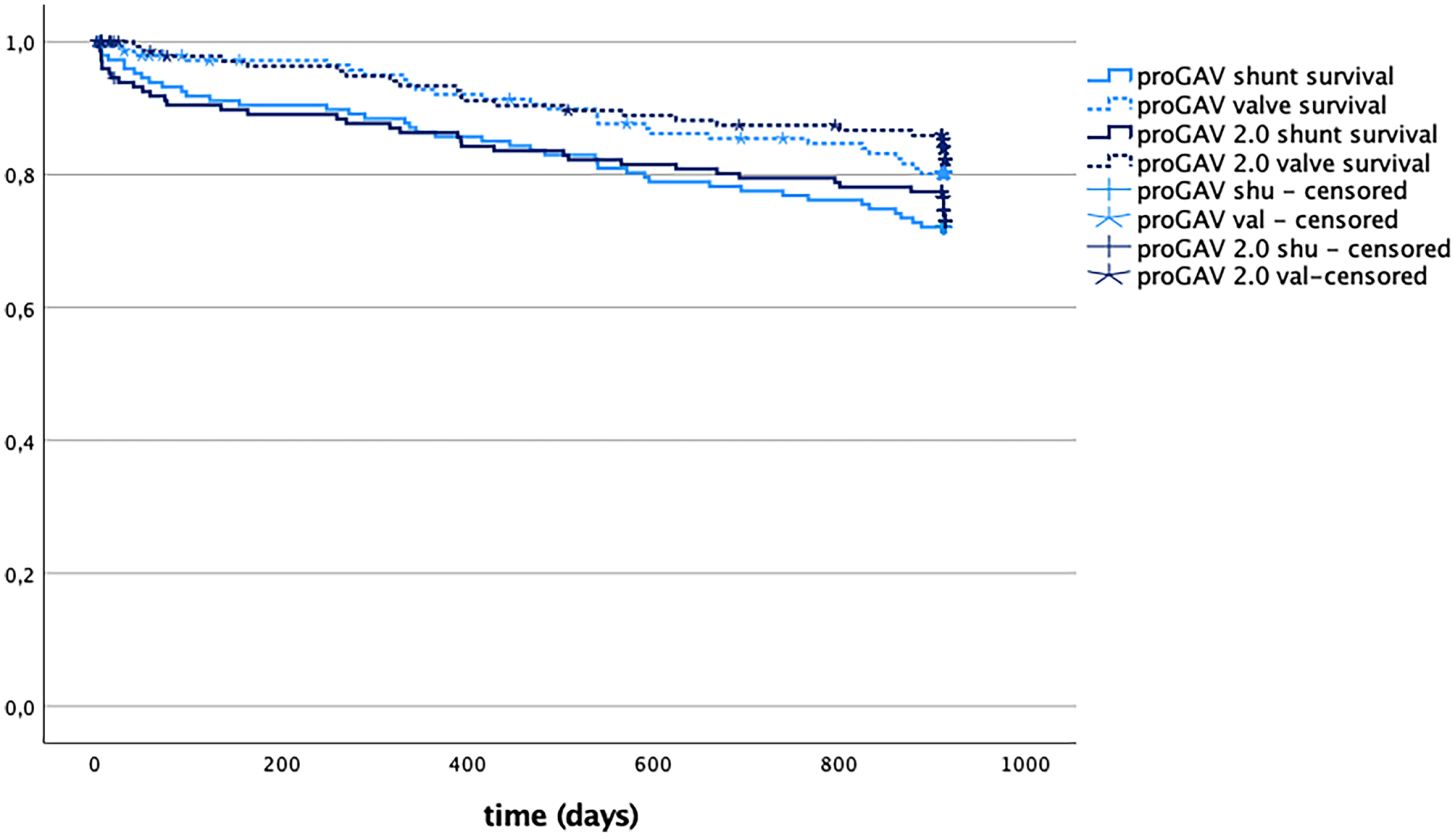
Kaplan–Meier curve showing shunt revision-free survival (proGAV: 72.1% *n* = 106, proGAV 2.0: 74.8% *n* = 110) and valve revision-free survival (proGAV: 81.6% n = 120, proGAV 2.0: 85.0% *n* = 125) after 30 months follow-up without showing statistically significant differnces comparing the groups

Over 2 years (July 2012–June 2014), 142 patients underwent 147 shunt operations with valve implantation and received a proGAV as a standard regimen (Table 1). Forty-nine (33.3%) of these were primary implantations and 98 (66.7%) were revision procedures. Within 30 months follow-up, 41 shunts had to be revised. Revision procedures were categorized into groups according to the part of the shunt system that was revised: ventricular catheter alone (*n* = 12; 8.2%), ventricular catheter and valve (*n* = 3; 2%), valve alone (*n* = 11; 7.5%), valve and distal catheter (*n* = 5; 3.4%), distal catheter alone (*n* = 2; 1.4%), and complete shunt revision (*n* = 4; 2.7%). Occlusion of the valve was considered as reason for replacement in 15 (10.2%) cases. In 4 cases, the shunt system was removed without immediate replacement, either due to infection (*n* = 2, 1.4%) or, in 2 cases, because the indication for further shunting was no longer given. In 106 cases, no revision surgery was necessary within the follow-up period, resulting in a shunt survival rate of 72.1%. In 27 cases, the valve was replaced, resulting in a valve survival rate of 81.6% (Fig. 4).

In comparison, 147 proGAV 2.0 were implanted in 142 patients during a 2-year period between January 2015 and December 2016 (Table 1). Forty-six (31.3%) implants were primary implants and 101 (68.7%) were secondary implants. Within the following 30 months, 37 of these systems were revised, specifically: ventricular catheter alone (*n* = 13, 8.8%), ventricular catheter and valve (*n* = 4, 2.7%), valve alone (*n* = 10, 6.8%), distal catheter alone (*n* = 3, 2.0%), complete shunt (*n* = 3, 2.0%). Blockage of the valve was considered the reason for valve replacement in 11 (7.5%) cases. In 4 cases, the shunt system was removed without immediate replacement, either due to infection (*n* = 3, 2.0%) or, in 1 case, because the indication for another shunt was no longer given. In 110 cases, no revision surgery was necessary within the follow-up period, resulting in a shunt survival rate of 74.8%. In 21 cases, the valve was replaced, resulting in a valve survival rate of 85.7% (Fig. 4). No statistically significant difference for shunt survival (*p* = 0.65) or valve survival (*p* = 0.46) were between proGAV 2.0 and proGAV group. The total number of shunt operations per patient during the follow up period was 1.34 ± 0.6 (range: 1 to 4) in the proGAV group and 1.32 ± 0.6 (range: 1 to 4) in the proGAV 2.0 group showing no statistical difference.

As shown in Table 2, the results of valve and shunt survival rates of “infants” (age: < 1 year) and “non-infants” as well as “PHHC” and “other diagnosis” were similar for each valve model and did not show a statistically significant difference among the groups either. However, there was a significantly better outcome in shunt survival (*p* = 0.03) and valve survival (*p* = 0.005) in non-infants compared to infants, regardless of diagnosis and valve model.

**Table 2:** Comparing the shunt- and valve revision-free survival rates between the different clinical subgroups in the 1st study cohort. Possible differences among the subgroups are evaluated with Log rank (Mantel cox) test

		Valve		Shunt	
		Survival rate	*p* value	Survival rate	*p* value
Age	Infant	75.3%	**0.005**	65.5%	**0.03**
	Non-infant	87.6%		77.1%	
Diagnose	PH HC	78.9%	0.11	69.7%	0.32
	Others	86.5%		75.7%	

### Length of valve implant duration

**Fig. 5:**
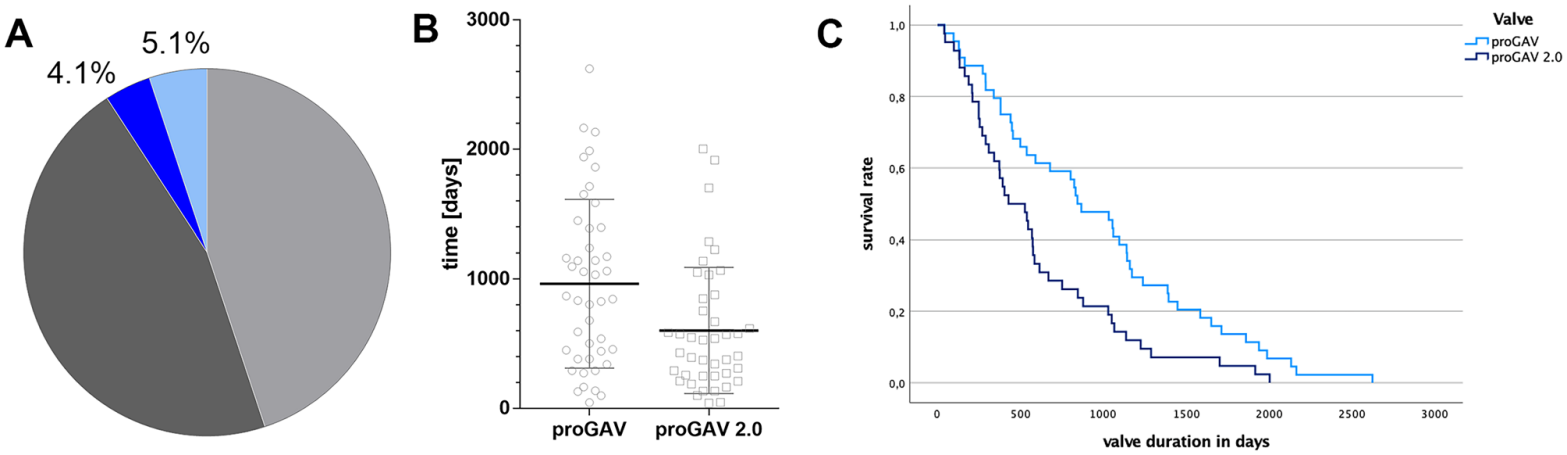
Patient cohort (*n* = 27), who underwent a valve-only-revision (**A**: proGAV: *n* = 12, 4.1%; proGAV 2.0: *n* = 15, 5.1%) together with another valve exchange in relation to the entire study cohort. **B** Mean valve implantation periods for all detected valve revisions (n=86) from 38 patients, respectively. The proGAV (*n* = 44) showed an implant duration of 961.9 ± 650 days implantation period being significantly longer than the proGAV 2.0 (*n* = 42; 601.4 ± 488 days; *p* = 0.007). **C** Kaplan–Meier valve revision-free survival curves ending at 0% by definition, that all valves were explanted in this study cohort. The revision-free survival rate at 12 months was 79.5% for proGAV and 61.9% for proGAV 2.0, at 24 months 59.1% for proGAV and 28.6% for proGAV 2.0 reflecting a significant prolonged survival for proGAV (*p* = 0.004)

In the second study the length of valve implant duration of patients who had valve-only-revision are evaluated (Table 3). This accounted for 27 patients of the cohort of the 1st study (proGAV *n* = 12 (n=1 during inclusion; n=11 during follow-up); proGAV 2.0: *n* = 15 (n=5 during inclusion; n=10 during follow up); Fig. 5A). For these patients, the mean implantation period was 1272 ± 698 days (range: 290 to 2622) for the proGAV, which was significantly longer compared to 744.5 ± 507.2 days (range: 46 to 1916 days; *p* = 0.03) for the proGAV2.0. Including additionally all valve only revisions in the entire study period and reviewing previous and subsequent procedures a total number of 86 valves were included for which implant duration times were available (Fig. 2). Similarly, 44 proGAV (mean valve duration: 961.9 ± 650.8 days; range: 44 to 2622 days) showed a longer implant duration compared to 42 proGAV 2.0 (601.4 ± 487.8 days; range: 41 to 2003 days; *p* = 0.007; Fig. 5B).

**Table 3:** Patient characteristics for the second group of patients

**Time period**		July 2012–June 2019	
**Number of patients**		38	
**Age at first surgery**		4.01 y ± 3.90	
**Sex**		f=16 m=22	
		**proGAV**	**proGAV 2.0**
**Number of valve revsions**		60	61
**Number of available valve implantation time**		44	42
**Valve implantat duration time**		961.9 ± 650.8d; range: 44d to 2622d ***n* = 44**	601.4 ± 487.8d; range: 41d to 2003d ***n* = 42**
** -Implant duration** * cohort from study 1 (n=27)*		1272 ± 698d range: 290 to 2622d ***n* = 12**	744.5 ± 507.2d range: 46 to 1916d ***n* = 15**
** -Implant duration** ** (inclusion criteria study 2)** * after valve only revision (n=38)*		1249.73 ± 183.5d; range: 97 to 2622d ***n* = 15**	670 ± 104.6d; range: 46 to 1916d ***n* = 23**
** -Implant duration** * previous valve surgery*		868.77d ± 580.7d; range: 44d to 2164d ***n* = 26**	414.42d ± 336.7d; range: 41d to 1066d ***n* = 12**
** -Implant duration** * after first valve revision*		414.5d ± 48.8d; range: 380 to 449d ***n* = 2**	771.17d ± 655.0d; range: 132d to 2003d ***n* = 6**
** -Implant duration** * after second valve revision*		164d ***n* = 1**	249d ***n* = 1**

Looking at the Kaplan–Meier curve analysis for all implants of the second study cohort (Fig. 5C), the estimated survival rate at 12 months is 79.5% for proGAV (*n* = 44) and 61.9% for proGAV 2.0 (*n* = 42), at 24 months 59.1% and 28.6% and at 5 years 13.6% and 4.8%, respectively, being significantly lower for proGAV2.0 (*p* = 0.004). As the inclusion criteria for the second study cohort was valve-only-revision and a subsequent valve revision the survival rate consequently becomes 0 in both groups.

## Discussion

Shunt implantation is still the most often applied treatment of pediatric hydrocephalus. Revision-free survival rates are representing surgical data for hydrocephalus therapy reflecting some of the complications, which might be caused during the chronic therapy. The possible complication of over- and underdrainage is addressed by newer shunt valve designs. In those anti-hydrostatic functionalities and adjustability are offering mechanisms to overcome long-term overdrainage and adapt the CSF diversion to the individual needs of the patient [20]. With the proGAV 2.0, the successor of the proGAV, a new generation of adjustable differential pressure valve with fixed anti-gravitational unit was introduced to specifically improve the adjustability of the valves. Since our department was using the proGAV for many years as standard valve treating pediatric hydrocephalus we switched to proGAV2.0 in 2014. This gave us the opportunity to compare the two different valve types within a retrospective historic comparison cohort study using a standard time period design for both groups. We were able to show that the overall revision-free survival rate of the shunt as well as valve was decent during a 30-month follow-up period and did not differ between the two groups. Similarly, not only the first revision as depicted in the Kaplan Meier analysis but also the total amount of shunt and valve revisions during follow up were similar between the two valve designs. In order to specifically investigate the valve implant duration time, we selected patients, who received a valve-only-revision and had another valve revision during the follow up period representing specifically a cohort, who experienced an isolated valve dysfunction. Since this cohort had a second valve explantation, we consider these patients at risk for valve dysfunction. In this cohort of patients, we observed a significant longer implant duration for the proGAV compared to the proGAV2.0, which holds true for the group of valves as defined in the inclusion criteria as well as for all the valves which were previously and subsequently explanted in the defined study period.

Among previous studies, Rhode et al. was one of the first studies to investigate the proGAV valve in the pediatric setting in a bi-center study. With a mean follow-up time of 15 months and valve and shunt survival rates of 88.7 and 75.5%, respectively, a good clinical outcome was demonstrated. Although, there was no significant difference between proGAV (92.1% and 85.7%) and proGAV 2.0 (91.2% and 84.3%) shown in our study after 15 months follow-up time, we were able to observe similar survival rates [21]. The results were confirmed by a larger single-center cohort, in which 203 pediatric patients with shunted hydrocephalus were treated [14]. Gebert et al. investigated the shunt and valve revision-free survival rates of 93 infants, showing lower rates at 12 months of follow-up with a shunt survival rate of 69% and the valve survival rate of 78% most likely due to the fact that infants are more prone to shunt revisions [5]. This is in line with our results from our current study that shunt survival rates between infants and non-infants are showing relevant differences being similar in proGAV as in proGAV2.0 [5, 14]. The higher risk for infants for shunt failure is partially based on a higher rate of shunt infections. Additionally, Alavi et al. investigated the effect of changing from different valve models to proGAV. The shunt revision-free survival rate of 54.7% and a valve survival rate of 81% after a mean follow-up time of 36.3 months in 46 patients studied [15]. A more recent paper was addressing for the first time the experience of the proGAV2.0 as compared to a fixed differential pressure valve with gravitational assistance (paediGAV). They found significantly lower amount of revisions in the proGAV2.0 compared to the paediGAV and the revision-free survival was comparable with our results, respectively [22].

In addition to comparing revision rates, our present study also aimed to investigate whether there was a difference in the length of implant duration periods between proGAV and proGAV 2.0. That was specifically investigated in patients at potential risk for valve dysfunction since they had a valve only revision and at least one other valve exchange during the study period. Through this work, it was found that the proGAV valve has significantly longer implant duration on average than the proGAV 2.0. This addressed 27 patients, 9.3% of the entire cohort of the 1st study part, but could possibly involve more patients, if all revised valves would be ivestigated. This subgroup of patients might be a sensitive cohort since multiple valve revisions were detected in the respective study period. In those patients, a different distribution of underlying reasons for hydrocephalus was seen compared to the entire cohort of the 1st study part. Specifically, post-hemorrhagic and post-infectious hydrocephalus occurred significantly more often and accounted for 66% of the valves in the second study group versus 36% in the remaining cohort. We suspect the reason for valve failure to be partial or complete valve occlusion by protein debris. This might lead to either underdrainage or to functional impairment of the adjustability of the valve. Since PHHC as well as PIHC is often related with persistent increased CSF protein levels, in which large number of different types of proteins are represented [23]. For this subgroup of patients, we confirmed this protein debris in the explanted by sending those for further investigation to the manufacturer and receiving the photo documented confirmation, accordingly. Further investigations are necessary and are currently on the way to assess this issue in a prospective study design. Nevertheless, the issue of valve occlusion was previously investigated more in detail by others, which elucidated that not only proteins like laminin, fibrin, and collagen were observed, but also cellular components like macrophages, monocytes, and astrocytes [24]. It remains not fully understood, how these connective tissue proteins are brought into the valve system, which is a matter of our further investigation. We hypothesize that the partial or complete valve occlusion may be the main reason also in our study cohort for isolated valve failure, and a subgroup of patients may carry a higher risk in this respect. Since the total lumen of the proGAV is larger than the one of proGAV2.0 it may be possible, that the functional failure by occlusion becomes simply relevant at an earlier time point if the inner valve lumen is smaller. Thus, we suggest to still use proGAV in patients with possible higher risk of occlusion (e.g., patients with PHHC or other causes for increased CSF protein levels or patients with repeated proven valve failure) in order to enable longer valve duration. This comes, however, along with accepting a less comfortable adjustment functionality in the proGAV.

Future investigations should focus on a more detailed analysis on the exact reason for valve failure and in occluded valves the CSF as well as inner valve debris together with patient characteristics should be investigated in more detail. Further insights may lead to development of better protective materials used for pediatric hydrocephalus valves to prevent occlusion in the future.

## Conclusion

Our historical comparative study between proGAV and proGAV2.0 could show that revision-free survival rates were found to be similar and at a decent rate for a 30-month follow-up period. Not only the shunt and valve revision-free survival rates but also the total amount of shunt surgeries per group showed similar values. However, looking at patients who underwent valve-only-revision and including all possible valve revisions during the study period in this subgroup of patients, we found a significant longer implantation times for the proGAV compared to the proGAV2.0, which may likely be related to valve occlusions. Future research should focus on detailed characterization of valve failure to improve material properties that prevent occlusion rates in long term.
